# Supplementary figures and images for: Intra-lineage microevolution of Wolbachia leads to the emergence of new cytoplasmic incompatibility patterns
Source: PLoS Biol. 2024 Feb 5;22(2):e3002493. doi: 10.1371/journal.pbio.3002493 (PMC10868858; doi:10.1371/journal.pbio.3002493)

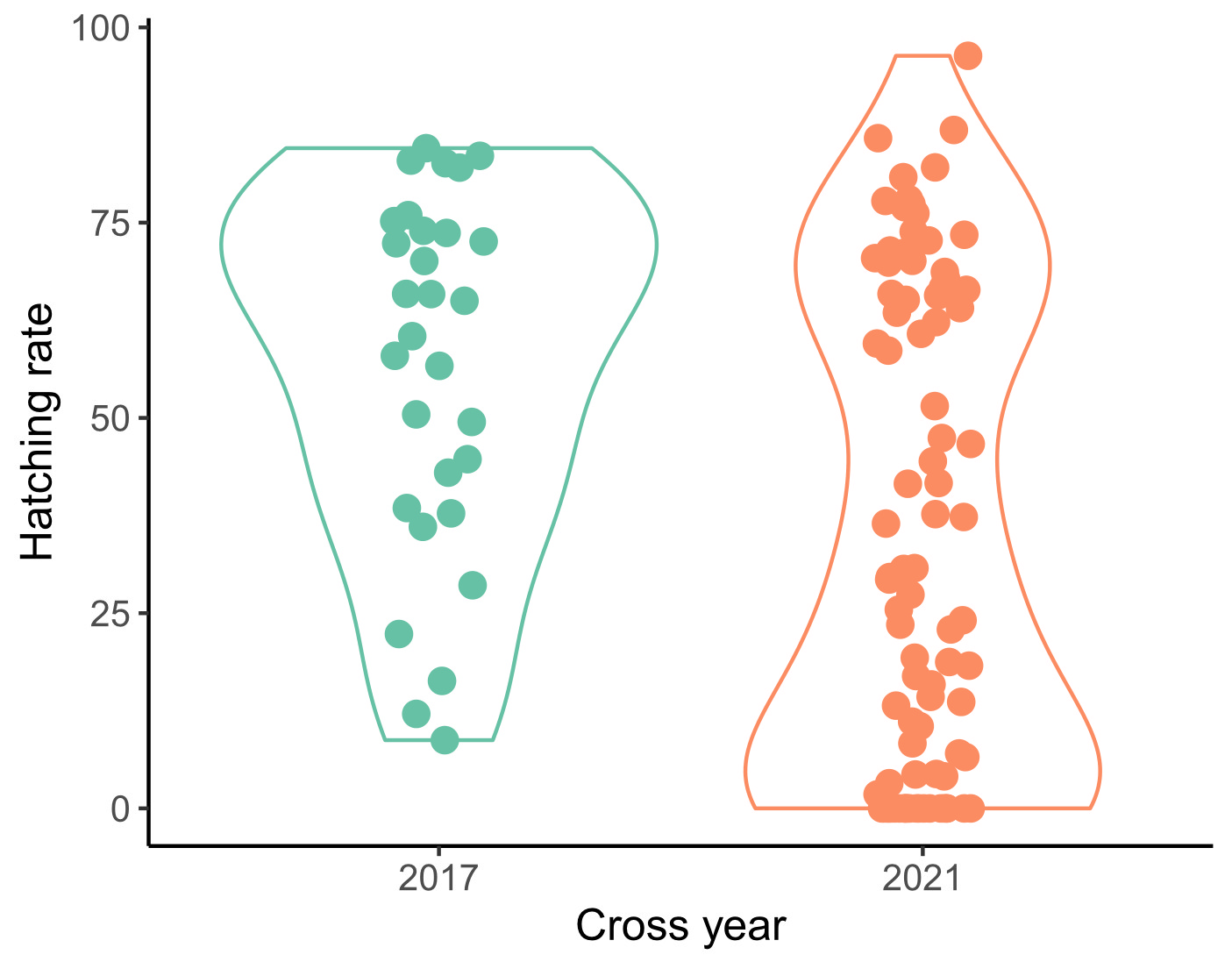

Supplement: S1 Fig — A notable difference is the existence of crosses with null hatching rate in 2021. Data supporting this figure are found in [41] (for 2017 Data), and in S2 Data (2021 Data). (TIF) [file pbio.3002493.s001.tif]

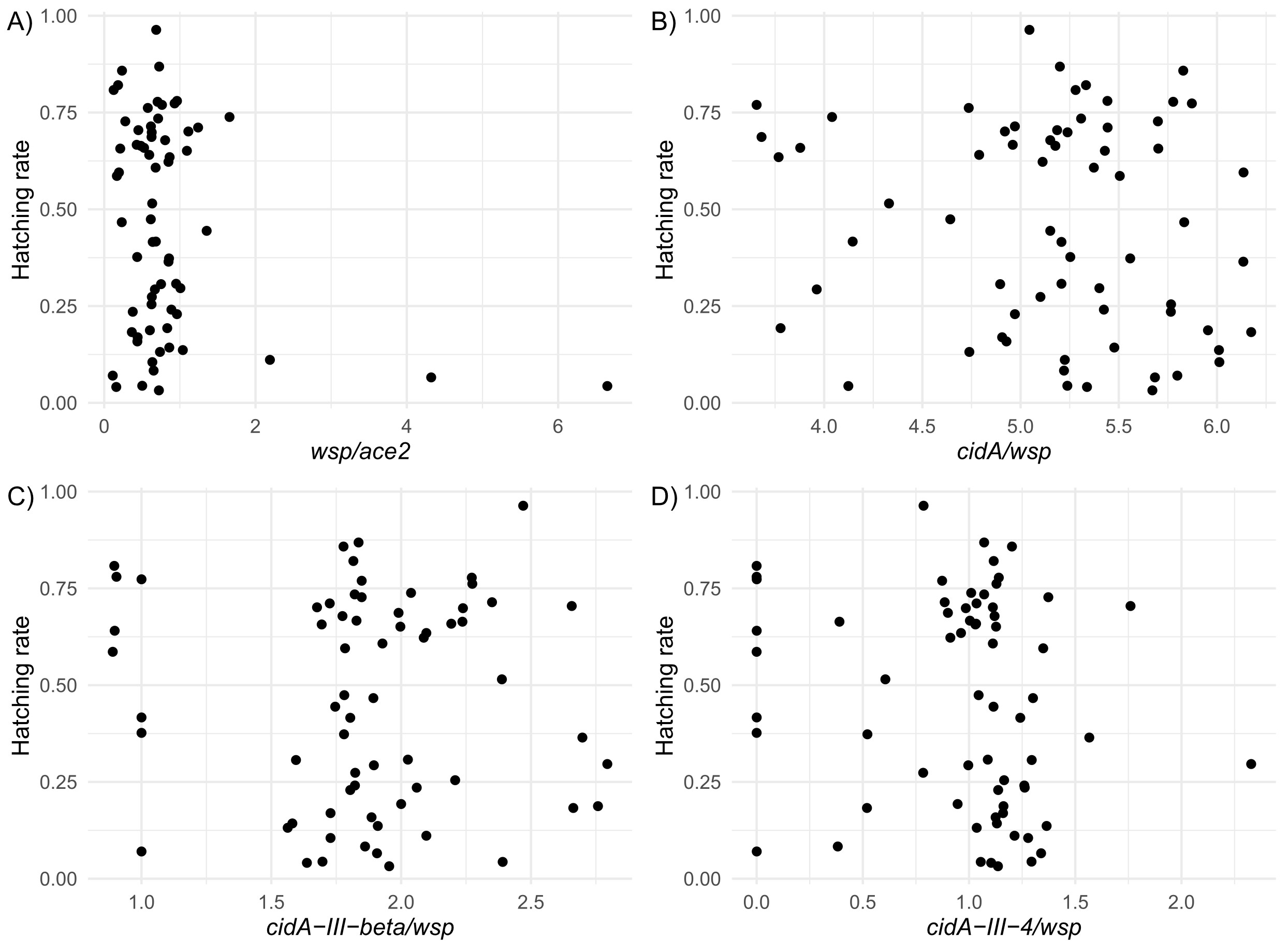

Supplement: S2 Fig — Hatching rates in relation to variations in: (A) Wolbachia infection level and numbers of copies of (B) total cidA, (C) cidA-III-β(2), and (D) cidA-III-16. None of these variations are significantly correlated with hatching rates (Spearman correlation coefficient, P = 0.49, 0.28, 0.94, and 0.16 for (A–D), respectively). Data supporting this figure are found in S1 Data. (TIF) [file pbio.3002493.s002.tif]

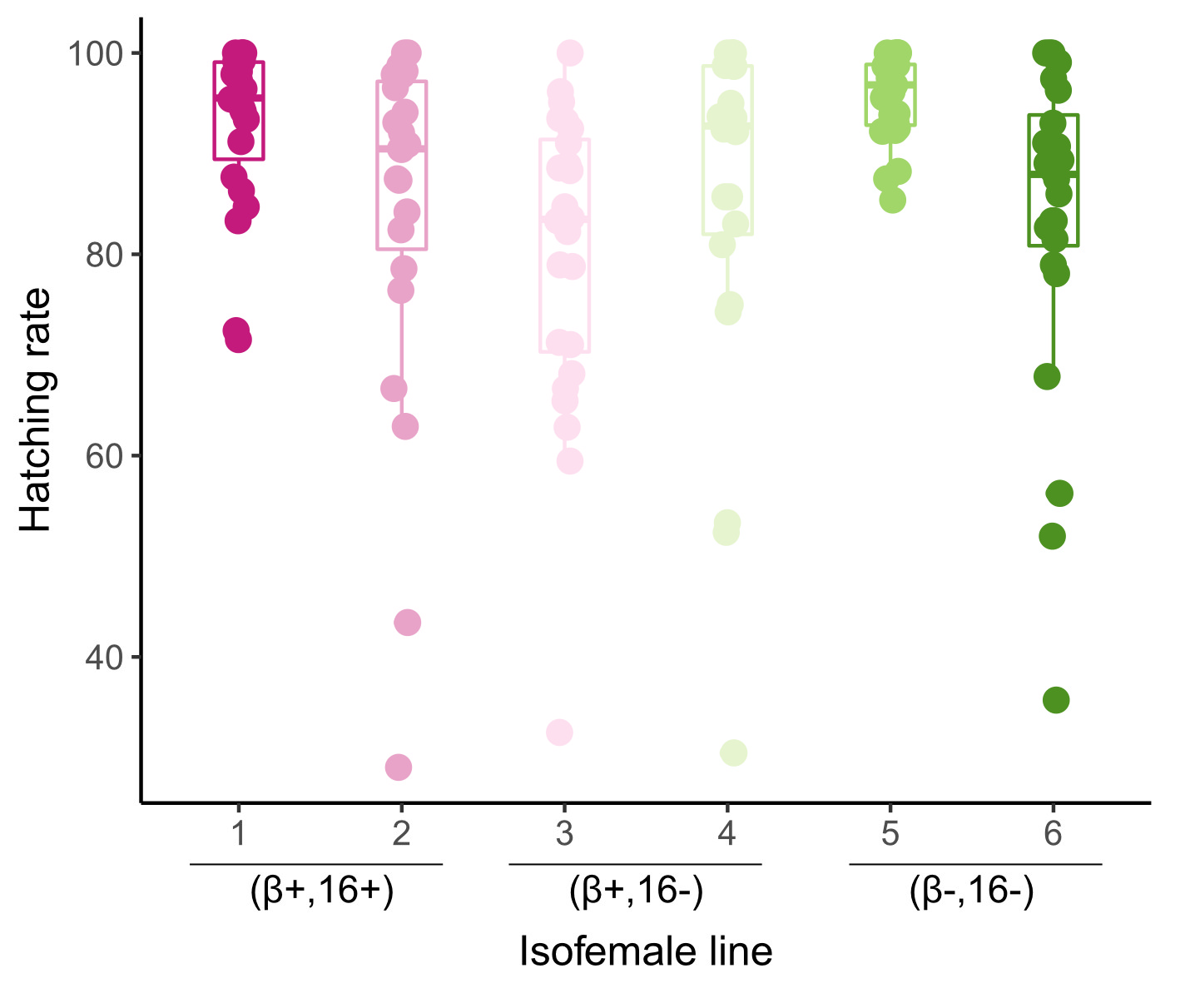

Supplement: S3 Fig — For each line, 20 to 30 egg rafts were collected and the hatching rate was measured. Data supporting this figure are found in S2 Data. (TIF) [file pbio.3002493.s003.tif]

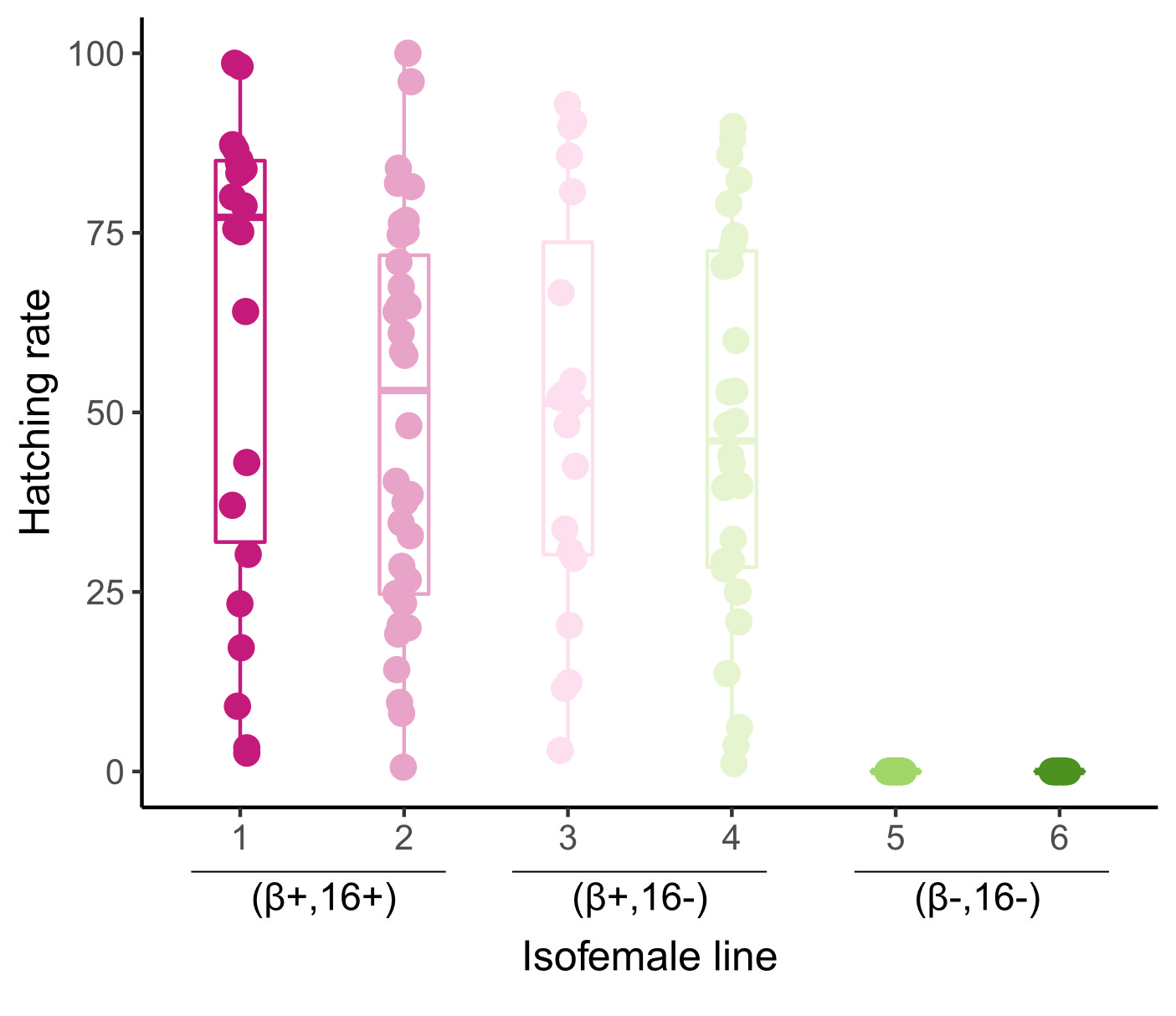

Supplement: S4 Fig — Data supporting this figure are found in S3 Data. (TIF) [file pbio.3002493.s004.tif]

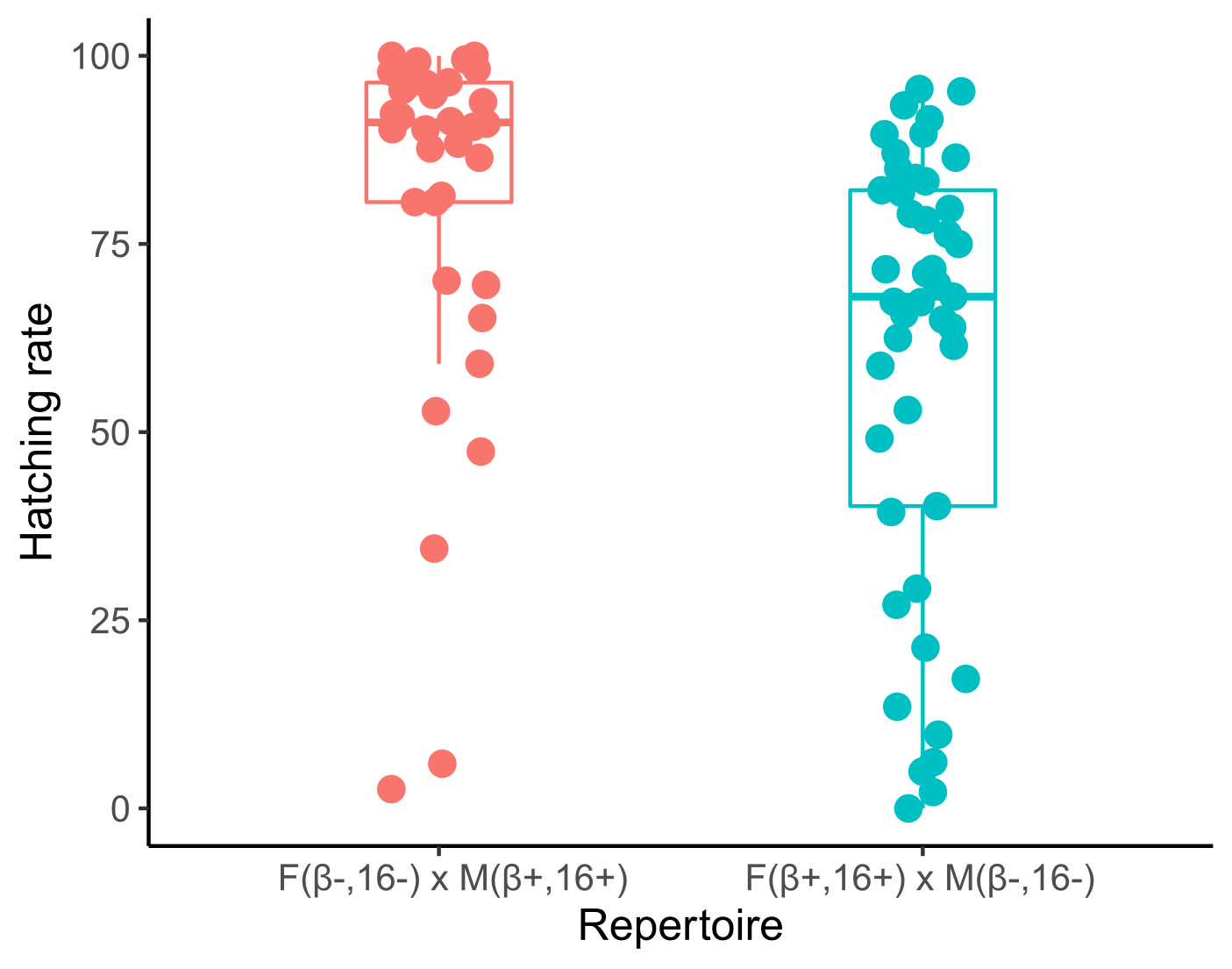

Supplement: S5 Fig — Data supporting this figure are found in S4 Data. (TIF) [file pbio.3002493.s005.tif]

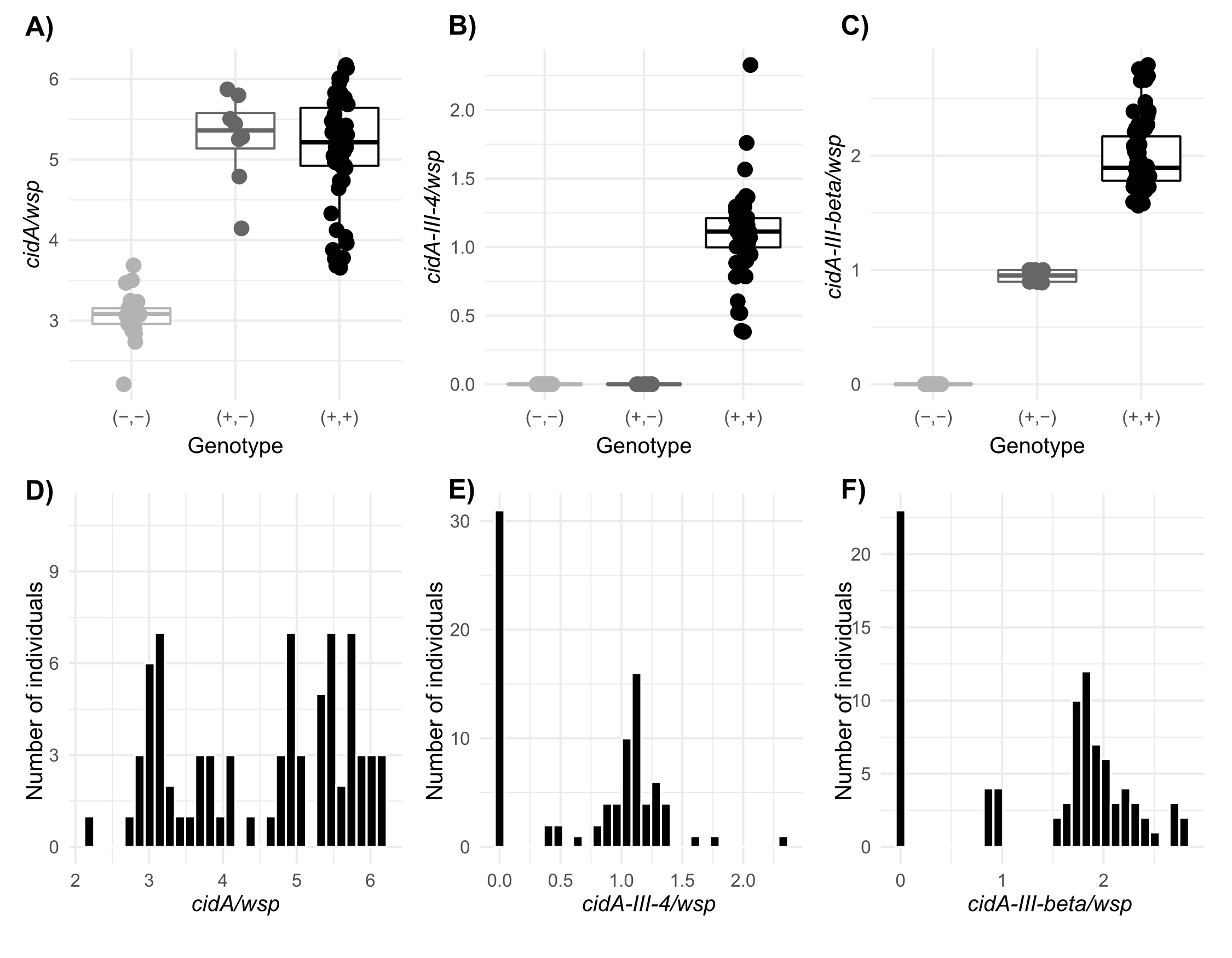

Supplement: S6 Fig — (A–C) show the numbers of copies of total cidA, cidA-III-16, and cidA-III-β(2) regions, respectively, in females with repertoires (β-,16-), (β+,16-), and (β+,16+). (D–F) show the distribution of cidA, cidA-III-16, and cidA-III-β(2) copy numbers. All histograms show either bi- or trimodal distributions. All copy numbers were quantified using quantitative PCR relative to the single copy Wolbachia gene wsp, on 23 (β-,16-), 8 (β+,16-), and 58 (β+,16+) adult females. Data supporting this figure are found in S1 Data. (TIF) [file pbio.3002493.s006.tif]

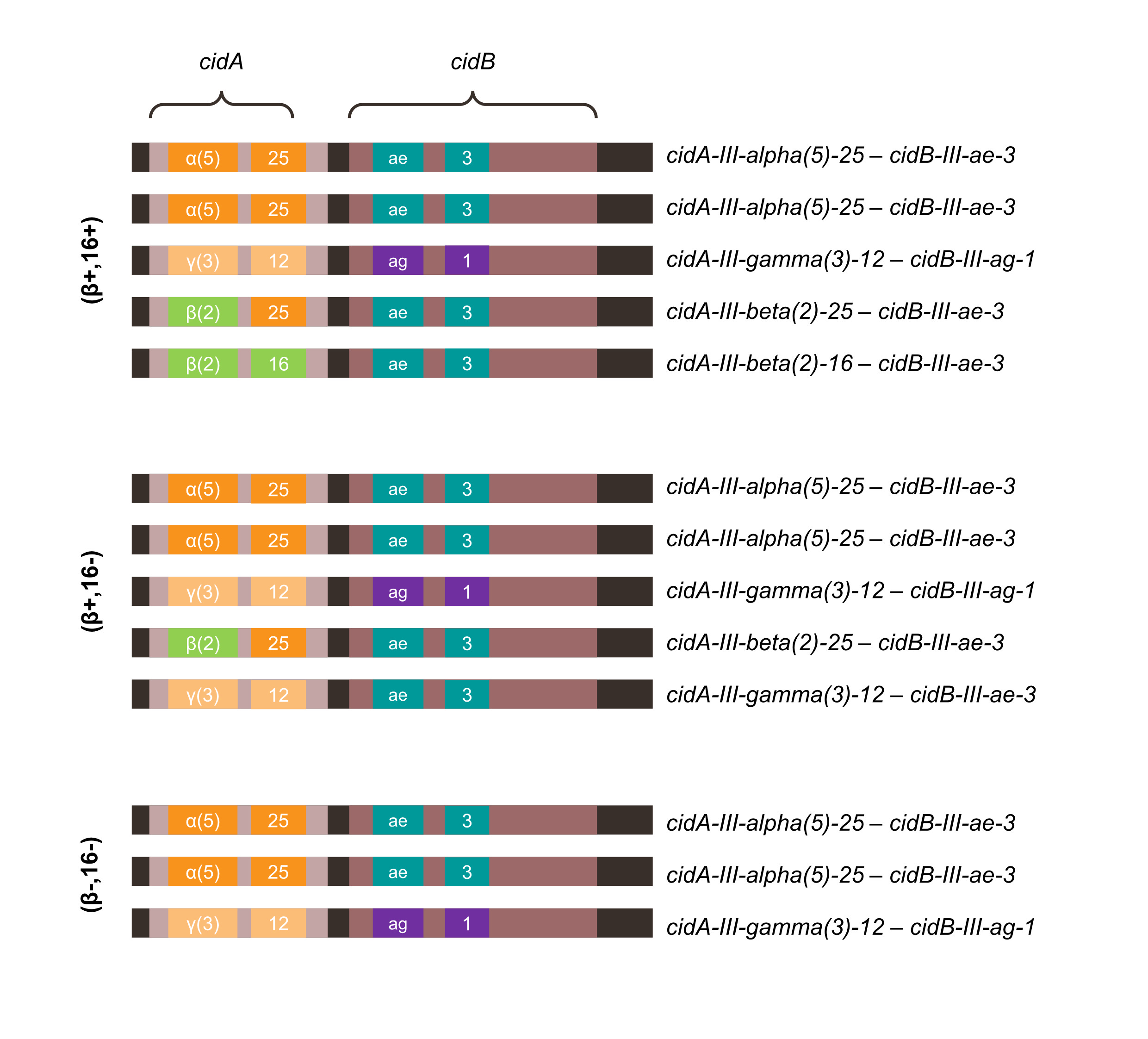

Supplement: S7 Fig — The cidA gene is represented in light purple and cidB in dark purple. Upstream and downstream variable regions are shown in color. Regions in green are the key studied regions: cidA-III-β(2) and cidA-III-16. The copy number of each tandem was deduced by combining the relative coverages in nanopore sequencing data with the qPCR data. (TIF) [file pbio.3002493.s007.tif]

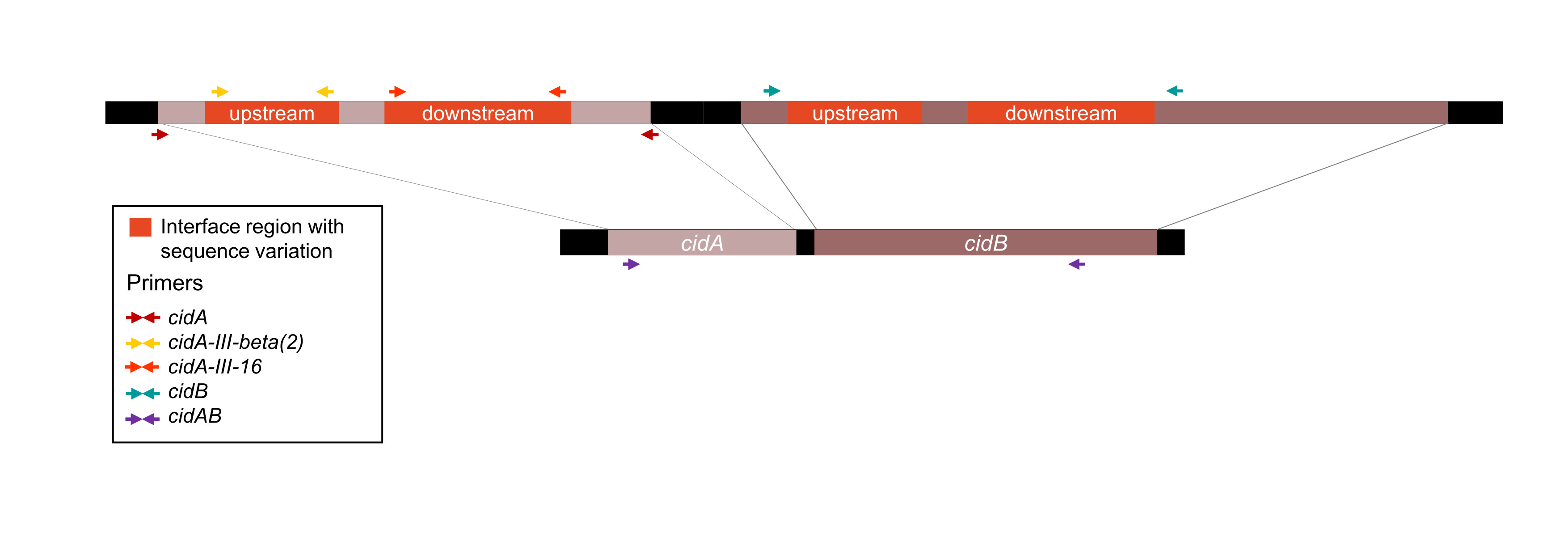

Supplement: S8 Fig — PCRs amplifying the full cidA gene (burgundy arrows), variable regions of the cidB gene (green arrows), and the cidAB tandem (purple arrows) were used for Nanopore sequencing of repertoires. PCRs specific to cidA-III-β(2) (yellow) and cidA-III-16 (orange) were used both in standard PCR and qPCR to determine the presence/absence of these regions. (TIF) [file pbio.3002493.s008.tif]

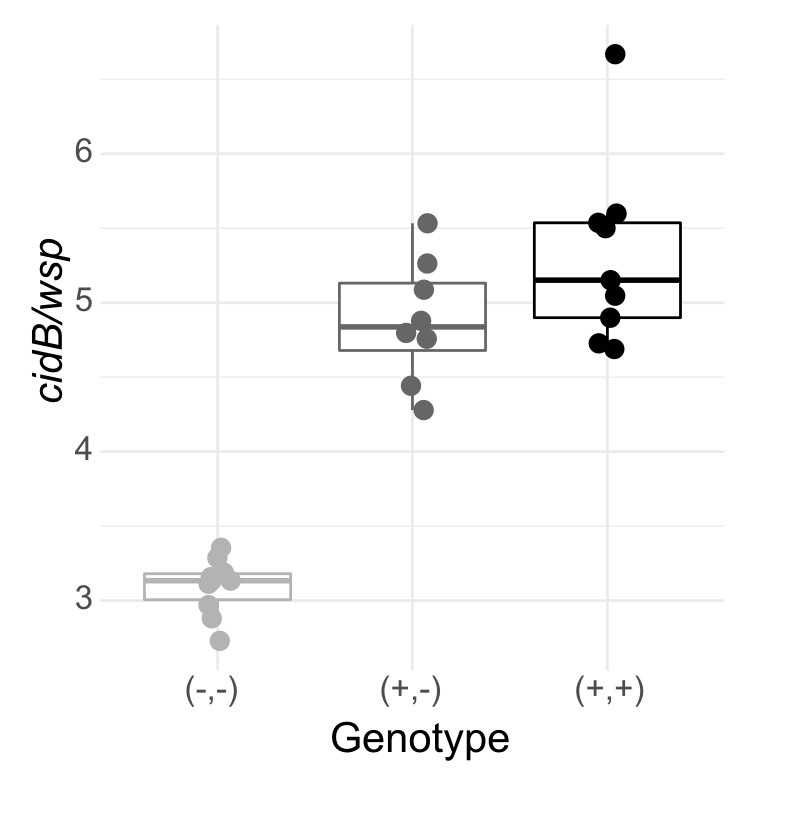

Supplement: S9 Fig — cidB copy numbers are similar to cidA copy numbers. (β-,16-) wPip has around 3 cidB copies, whereas (β+,16-) and (β+,16+) have around 5 cidB copies. All copy numbers were quantified using quantitative PCR relative to the single copy Wolbachia gene wsp. Data supporting this figure are found in S1 Data. (TIF) [file pbio.3002493.s009.tif]

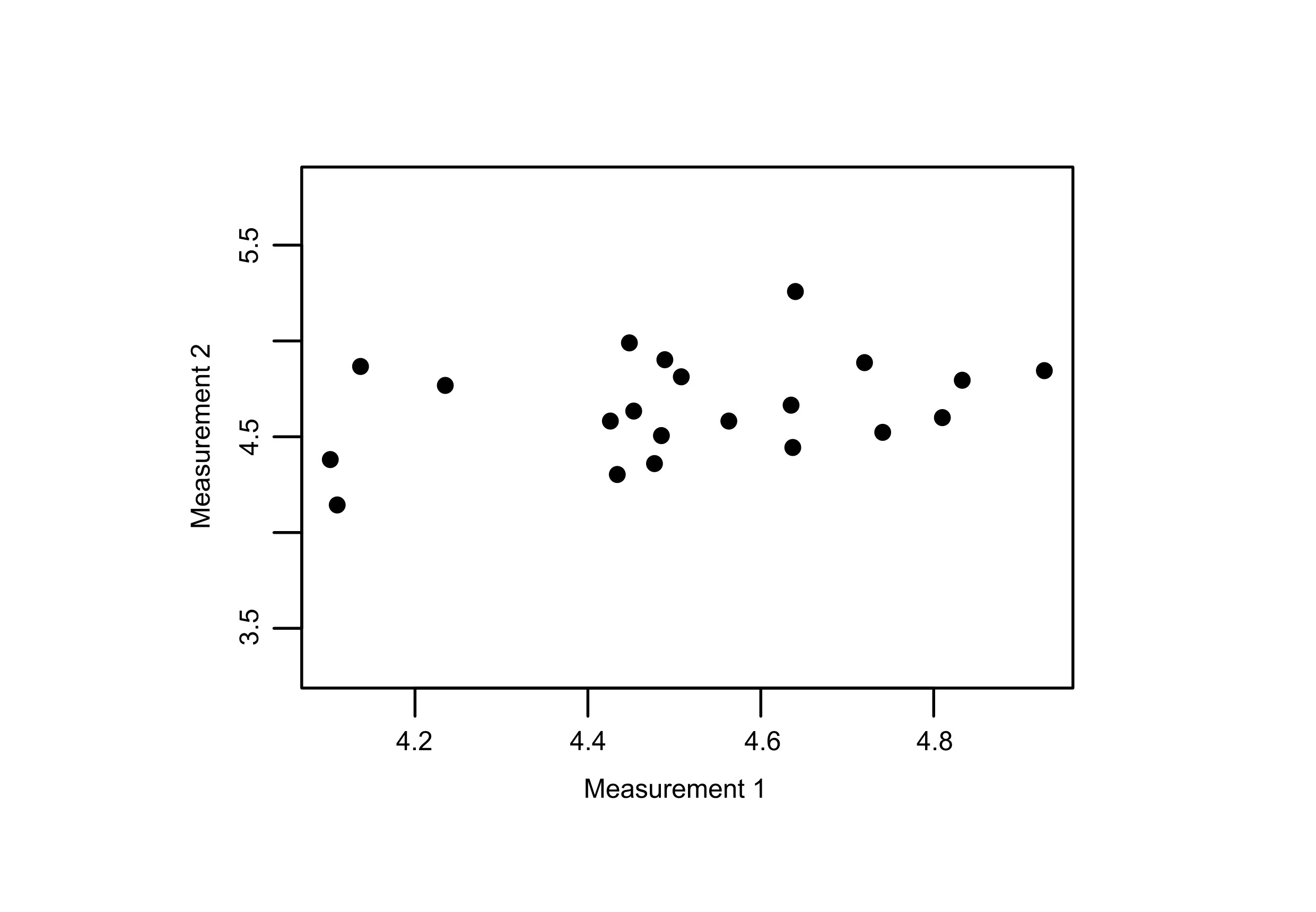

Supplement: S10 Fig — cidA copy numbers from the same individuals were measured twice (measure 1, measure 2) in 2 distinct qPCR runs. While all measurements are around 5 copies, there is no significant correlation between the first and the second measurement. Data supporting this figure are found in S5 Data. (TIF) [file pbio.3002493.s010.tif]
