# Supplementary material for: Intra-lineage microevolution of Wolbachia leads to the emergence of new cytoplasmic incompatibility patterns
Source: PLoS Biol. 2024 Feb 5;22(2):e3002493. doi: 10.1371/journal.pbio.3002493 (PMC10868858; doi:10.1371/journal.pbio.3002493)
Supplement: S1 Table — Nanopore sequencing of cid PCR products gave identical repertoires for 4 distinct Istanbul individuals. (DOCX) [file pbio.3002493.s011.docx]

| ***cidA*** | ***cidB*** |
| --- | --- |
| *cidA-IV-alpha(5)-1*  *cidA-IV-alpha(5)-2*  *cidA-IV-delta(1)-1*  *cidA-IV-delta(1)-2*  *cidA-IV-gamma(5)-1*  *cidA-IV-gamma(5)-2* | *cidB-IV-a1*  *cidB-IV-a2*  *cidB-IV-b1*  *cidB-IV-b2*  *cidB-IV-d1* |
