# Supplementary material for: Intra-lineage microevolution of Wolbachia leads to the emergence of new cytoplasmic incompatibility patterns
Source: PLoS Biol. 2024 Feb 5;22(2):e3002493. doi: 10.1371/journal.pbio.3002493 (PMC10868858; doi:10.1371/journal.pbio.3002493)
Supplement: Attachment — Submitted filename: Namias_et_al2023_reviews.docx [file pbio.3002493.s020.docx]

Dear Editor,

Thanks for helpful insights. We followed the comments by the Academic editor and 3 anonymous reviewers and (i) made complementary experiments, with new crosses to analyze hatching rates variations in intra-line situations, and qPCR analyses, showing that within-group variability in copy numbers is likely due to qPCR errors, rather than true differences; (ii) shortened and clarified multiple sections, along with greatly improving the figures and adding proper statistical analyses. The context and previous studies are now more clearly stated, as required by reviewers.

Detailed answers to Editor and Reviewer’s comments are found below.

Dear Dr Namias,

Thank you for your patience while your manuscript "Intra-lineage microevolution of Wolbachia leads to the emergence of new cytoplasmic incompatibility patterns" was peer-reviewed at PLOS Biology. It has now been evaluated by the PLOS Biology editors, an Academic Editor with relevant expertise, and by three independent reviewers.

You’ll see that reviewer #1 is very positive about the paper, but has a long list of presentational improvements and requests for clarification; some of these may involve new data. Reviewer #2 says it’s interesting, but currently better suited to a specialised audience. His/her main concern is expressed in “point 1,” which is a list of missing aspects; while these would clearly improve the study, we think that supplying all of them may not be reasonable for a Short Report (see Academic Editor's comments below). Her/his “point 2” complains about the frustratingly arcane nomenclature (I totally agree!) and makes some presentational suggestions. Reviewer #3 does not comment on the advance, but has several requests for methodological clarity, wants some formal stats, finds the abbreviations confusing, and has a number of presentational suggestions.

IMPORTANT: Given the divergent opinions on this paper, I discussed the reviews with the Academic Editor who kindly provided some additional guidance which you may find helpful when deciding how to revise the paper, and which you should address directly:

"An issue pointed out by the reviewers, and that I agree with, is that the paper is difficult to read and would need to be re-written to a certain extent.

We tried to simplify the paper by re-writing it in parts, and notably shortened the discussion section.

"Another common point is that some of the data that is discussed should be presented, as for example, compatibility data between the lines in the beginning and when they noticed that there was a change. Also, self-compatibility data is missing, and would help to understand better the results. Self-compatibility data may have to be generated and may be difficult to obtain in a short time.

The initial compatibility data between lines, and the time when a change was noticed are detailed in lines 90-96 of the introduction. We also gave details on the hatching rates at those times in the first paragraph of results section.

We made the intra-line crosses for the six isofemale lines which we established for the study. Results are now available in Table S3 and show that hatching rates variability exists even in intra-line crosses. HRs ranged from 85% to 100% and from 35% to 100% in the two (β-,16-) intra crosses, from 32% to 100% and from 30% to 100% in the two (β+,16-) intra crosses, and from 29% to 100% and from 71% to 100% in the two (β+,16+) intra crosses respectively. Although some crosses exhibited extreme hatching rates than others, there is no significant difference in variance among all those intra-line crosses.

However, although there was variability in intra-line crosses, this variability was significantly lower to that observed in the “compatible” Slab x Ist crosses. These results are now detailed in lines 174-179 (Levene test, *p* < 0.0001).

"I think the fitness of the new variant is not an essential aspect of this paper as a short report. But reviewer #2's point 1 request may be related to how the paper is written and presented. The abstract ends on speculation on evolutionary and ecological dynamics of CI and trying to project these findings in a lab population to natural populations dynamics. The fact that these genes can be easily lost, is very interesting with many possible consequences. But maybe the main ecological and evolutionary conclusions should be presented more as speculation and less as conclusion. I think the authors don’t discuss for example why are these genes maintained in Wolbachia in a natural population and how different is a natural population and an isofemale line with one founding Wolbachia.

We changed the abstract to make the final sentence more speculative.

We think that there could be a stronger selection for the maintenance of *cid* in natural populations, because a diversity of repertoires coexists in the field. Higher diversity of *cidB* likely selects for the maintenance of a high *cidA* diversity. We had made this point in the section “Rapid evolution” of the discussion. We tried to make it clearer.

"For me the strength of the paper is more on having a specific phenotype change in CI associated with a specific change in genotype in CI genes. This is actually a very strong case, supported on Wolbachia genetics, for how CI works. All the previous great work is mostly supported by transgenes assays. More, I would argue, which the authors don’t, that the findings are a great support for the toxin-antitoxin model. A very specific recombination between two CidB is only rescued by a recombination between two CidA that seems to restores sequence compatibility.

Thanks for the advice, we should have said it clearly! It is now said in the abstract, and in the discussion.

"Overall, I agree with the major revision and see how the authors respond to the comments. I would stress:

1) the re-writing to make the paper easier to understand

2) presenting more of the data that is discussed

3) more clarity in what is shown and possible ecological and evolutionary dynamics consequences."

We re-wrote part of the paper, and hope to have improved the ease of reading. We made intra-line crosses and made the data available in Table S3, and we clarified the starting point of the study by making clear which crosses were previously published, and which were made for the current study (lines 111-117).

We also suggested the evolutionary dynamics in natural populations rather than affirming them.

In light of the reviews, which you will find at the end of this email, we would like to invite you to revise the work to thoroughly address the reviewers' reports.

Given the extent of revision needed, we cannot make a decision about publication until we have seen the revised manuscript and your response to the reviewers' comments. Your revised manuscript is likely to be sent for further evaluation by all or a subset of the reviewers.

REVIEWERS' COMMENTS:

Reviewer #1:

Wolbachia bacteria are widespread in insect populations and can influence their reproduction through cytoplasmic incompatibility. Culex pipiens mosquitoes have diverse Wolbachia infections which coincides with a variety of crossing types, where different Wolbachia variants induce cytoplasmic incompatibility with some variants but not others. While the genes responsible for cytoplasmic incompatibility have been identified, the basis of this crossing type diversity is not fully understood and it is unclear how it evolved. Here, the authors describe the emergence of a Wolbachia variant with a new cytoplasmic incompatibility type over a very short period (<4 years) in a laboratory colony. This came from an observation that a cross between two previously compatible laboratory lines (Slab and Ist) were now partially incompatible, with around 25% of females in this cross producing zero viable offspring. They then established isofemale lines and performed sequencing to show that this was due to the loss of a variant of the CidA gene, which in the female is responsible for restoring compatibility with Wolbachia-infected males that have a different crossing type. Their experiments clearly demonstrate that these females have lost their ability to restore compatibility rather than the males evolving a new crossing type. This study appears to be the first case where the evolution of crossing patterns has been observed in real time and linked directly to a change in genotype.

Thanks, this was a well-phrased summary of our work, we used part of those sentences in the main text!

The paper is a significant advance in our understanding of Wolbachia evolution and has implications for mosquito control programs which rely on the release of Wolbachia-infected mosquitoes to induce cytoplasmic incompatibility to either suppress or replace local mosquito populations. While the paper is well written and the experiments are robust, I have several suggestions below:

Figure 1 shows outcomes from a cross between Slab females and Istanbul males then separates hatch rates by genotype of the females. The hatch rates for the compatible crosses are highly variable - is it possible that these lines are partially incompatible? No data for within-strain crosses (e.g. Slab x Slab) are shown but the hatch rates for these would likely be much higher and more consistent. This is also the case for the data in tables S3 and S4 where within line crosses were not completed or shown. While this doesn't affect any of their conclusions it's a shame that these were left out as it would help to confirm whether these variable hatch rates were due to cytoplasmic incompatibility, fitness costs or some other reason.

We added a supplementary table (table S3) showing intra-line compatibility outcomes for the six isofemale lines established for the purpose of this study. These results show that hatching rate (HR) variability also exists in intra-line crosses. Results from the Sicard et al. 2021 mBio paper give a further argument with intra-line crosses of the Slab-TC and Ist-TC lines (Slab and Ist lines with no *Wolbachia* infection due to a tetracycline treatment) show a high HR variability (see Figure 1 below). This shows that variable HRs exist in *Culex* even without a *Wolbachia* infection, suggesting a loss of fertility in these strains, probably due to a prolonged lab rearing due to inbreeding (e.g. Ekechukwu et al, G3 2015 in *Anopheles coluzzii*) rather than a *Wolbachia*-linked compatibility issue.

Figure 1 : Hatching rates (HR) distribution in intra-line crosses between Ist-TC mosquitoes, i.e. Wolbachia-free mosquitoes of Ist genetic background. HR are highly variable, in a Wolbachia-independent way.

However, we found that hatching rates were more variable in “compatible” Slab x Ist crosses than in intra-line crosses, suggesting that these lower hatching rates could be due to cytoplasmic incompatibility, as previously suggested by Sicard et al, mBio 2021. This is now described in lines 175-180.

For future work, have the authors considered performing backcrosses to test whether these patterns are influenced by the host genetic background, given that the two lab populations have different origins?

Previous work showed that, in *Culex pipiens*, on/off compatibility (hatching *vs* no hatching at all) patterns were preserved by introgressing a given *Wolbachia* in a distinct genetic background (e.g. Atyame et al, Mol Ecol 2011, Sicard et al, mBio 2021).

Introduction - Are Istanbul females compatible or incompatible with Slab males? This would be important background information

This cross is incompatible, we added it in the introduction, lines 95-96.

Lines 98-99 - This observation is the basis of the paper, but there is no data provided and the authors only cite a paper from 2006 in the previous sentence. Please provide or reference the historical data showing that these crosses were compatible from 2005-2017 and provide data on the incompatible crosses observed in 2021. It is unclear if the data in the paper are from this initial observation or if these experiments were done later.

We maintain these two isofemale lines at the lab since 2005. Compatible crosses between Ist males and Slab females were previously published: (i) in Duron, Mol Ecol 2006, based on crosses from 2005 (33 compatible egg rafts, HRs not computed at that time) and (ii) in Sicard et al, mBio 2021, based on crosses from 2017 30 compatible egg rafts with hatching rates ranging from 8 to 84%). Finally, crosses used in this study were done in 2021.

We made that clearer in lines 111-117.

Line 114 - This section is very brief and lacks context. Is this referring to the data shown in figure 1 or is this a separate experiment? If it's the latter, I would suggest providing the data and also including data prior to 2017 for comparison.

See the answer above, we hope we made that part clearer now.

Line 122 - Given that only four individuals were tested from the Ist line is it plausible that there is some undetected variation in cidA and cidB? And if so, could this be contributing to the variability in hatch rates within the compatible crosses in this study? Or perhaps there is additional variation in cidA in Slab females that is contributing to partial CI (as only four compatible individuals were sequenced)?

Yes, we may have missed some variability in repertoires, especially in terms of total variants copy numbers (which requires a qPCR). Yet, variations in HRs are observed in intra-Slab line crosses (as mentioned above), and in crosses between *Wolbachia-*free mosquitoes, meaning that this variability can occur even without Ist-repertoire variability.

Here we focused on the on/off compatibility (which is perfectly explained by the presence/absence of specific variants in the *w*Pip genome), rather than on hatching rates variations, which might be explained by nuclear background and environmental factors.

Line 161 - Is it correct that qPCRs were done in the females only? Given the lack of correlations here it seems likely that this variation in hatch rate is being driven by the male (such higher expression of cidb leading to partial cytoplasmic incompatibility).

Yes, qPCRs were only done on Slab females. Some variations could indeed be driven by the male in a *Wolbachia*-related manner. We could not check for such male effects with our protocols: we isolated females for egg laying, ensuring a clear mother identification, but we could not know which male had fertilized them. We could try to measure the influence of males in another study, but isolated pair crosses are difficult to achieve in *C. pipiens*. Fertility variations in either males or females could also be due to the long-time rearing in the lab, as aforementioned.

Line 214 - The (Β+,16+) female x (β-,16-) male cross has substantially lower and more variable hatch rates than the (β-,16-) Female x (β+,16+) male cross. It's a shame that within-genotype crosses have not been included as a control because this may reflect partial cytoplasmic incompatibility.

Within-genotype crosses are now accessible in Table S3.

We had noticed this dissymmetry between (+,+)/(-,-) and (-,-)/(+,+) crosses. We kept the lines and intended to backcross these mosquito lines to put them in similar nuclear backgrounds before studying fitnesses.

Line 216 - In future work, it would be interesting to see how the genotypic frequencies change in the original Slab line given that (β-,16-) is at a frequency of 0% in 2005 and around 25% when the isofemale lines were created. Based on the authors' hypotheses in the discussion it would likely continue to increase.

Yes, it would be really interesting to see if having more variants is costly *per se*! We could not test these hypotheses right away, as this would require to put both *Wolbachia* in a similar nuclear genetic background to measure the sole effect of the presence/absence of a variant. Such backcrosses experiments last for several months.

Line 297 - Since only 4 individuals were tested isn't it possible that variation in cid repertoires in Ist males has gone undetected?

Yes, we may have missed some variability in repertoires, especially in terms of variants copy numbers. This could contribute to the variation in hatching rates in ‘compatible’ crosses, see previous answer to “line 122”.

Line 314 - Which dataset is this referring to? I can't see any 2017 data in the paper- please provide it or cite a paper if it has been published previously

We now made that clear in the introduction line 93, and in the results section (lines 111-117): the 2017 data mentioned are the data from Sicard et al, mBio 2021.

Line 320 - While the repertoires must all be at least partially mutually compatible, there is a clear difference in hatch rate between the two reciprocal crosses which may suggest partial incompatibility. There is also no data for within-repertoire crosses so it is unclear if they are fully self-compatible.

See answers above (First comment of reviewer #1 and answers to “line 214”)).

Line 439 - Please provide more information about how these populations were maintained prior to experiments. Were they set up as isofemale lines when they were established in 2005, or was this done later?

Slab and Ist isofemale lines were set up as isofemale lines in 2005, and we established six new isofemale lines (two of each genotype) in 2021, for the present study. This is now mentioned clearly in the ‘isofemale lines’ section of the methods. All isofemale lines were reared in the same insectarium conditions, noted in lines 445-448.

Line 454 - How did you ensure that mosquitoes were unmated?

We followed the protocols from Duron et al, Heredity 2006 and recently confirmed in Bonneau et al., 2018 PloS pathogens: whenever a female is unmated, there is no embryo in any egg within a raft, which is clearly visible on a slide with a binocular magnifier. In incompatible crosses, you always observed a portion of the eggs within a raft that develop to various extents.

Line 459 - This should be a proportion (number of larvae divided by number of eggs), not a ratio

Thanks, this was an English mistake, which we now corrected.

Line 464 - How is a CI and non-fertilized egg distinguished?

In case of a non-fertilized egg, there is no embryo, while CI results in an aborted embryo clearly visible inside the egg with a binocular magnifier.

S3 table - for crosses with B-,16- females, were all hatch rates zero?

Yes!

Reviewer #2:

Cytoplasmic incompatibility (CI) is a remarkable phenomenon found in insects and other arthropods that is caused by bacteria that are transmitted from females to their offspring, by far the most common of which is Wolbachia. In the simplest form of CI, uninfected females produce few or no viable offspring upon mating with infected males, and as a result, infected females have a strong advantage over uninfected ones. There is great interest in using CI microbes to control insect pests and disease vectors and the recent discovery of the genes that cause Wolbachia CI has transformed the field. Wolbachia CI is caused by diverse pairs of genes that appear to function as a toxin (in sperm) and an antidote (in eggs). But we don't yet have a good understanding of how these genes actually work. How does the toxin damage sperm? How does the antidote counteract the toxin? How specific is the interaction between toxin, antidote, and host target? Finally, how do new toxin-antidote

combinations evolve?

The authors have been studying these questions in Culex pipiens mosquitoes. Besides its public health importance, Culex is an important model for studying CI because it is infected with diverse Wolbachia strains that exhibit a bewildering combination of compatibility/incompatibility types. Over the past decades, the authors have made a great deal of progress in trying to understand the 'CI code' underlying these combinations by carefully rearing and crossing various isofemale mosquito lines. Here they report that in 2021, they discovered the spontaneous emergence of a new (in)compatibility type in their lab stocks: some females from the 'Slab' line are now incompatible with males from 'Iist' line. They show that this is a result of recombination that has generated a new antidote gene sequence variant.

In my opinion, this is an interesting discovery because it shows and captures rapid evolution of Wolbachia CI types. It also helps contribute to our understanding of the logic

underlying how CI works. But I have a number of concerns with this manuscript. I hope my comments and suggestions help strengthen the paper and project.

1. I feel that the paper at this stage would be more appropriate for a more specialized audience. As the authors themselves discuss, the generality and ecological relevance of this result is not clear, as the new CI variant is a recently isolated lab mutant.

No, this variant is not a lab mutant: the recombinant β region, essential for compatibility with Ist males was present in the “initial” Slab line, and was lost in the cage since.

Also, its new incompatibility is cryptic, as it is only exposed in crosses to a different line. To be able to generalize this result, it would be useful and interesting to study the life history of this variant in more detail, as this would help predict its evolution. How does its fitness compare to the ancestral types with which it is co-segregating? Would it persist in 'Slab' experimental populations?

In *Culex* mosquitoes, incompatibilities are always a matter of specific crosses (line A x line B), as CI patterns are highly variable (e.g. Duron et al, Mol Ecol 2006, Atyame et al, Plos One 2014 for extensive characterizations of CI patterns in *Culex*). The recombinant β region studied here (and required in the Slab females to be compatible with Ist males) was found in natural populations of Tunisia and Italy (Bonneau et al, Nat Com 2018, Bonneau et al, Mol Ecol 2019). Indeed, all females from distinct mosquito lines bearing a *cidA* variant with a β region are able to rescue Ist males (Bonneau et al, Nat Coms 2018, Mol Ecol 2019), showing that the pattern we describe here is more general than the specific Slab x Ist cross. We made that point clear in the “recombination as the key to the emergence of CI patterns” section, in the discussion (starting line 392).

Changes in repertoire within a line may cause intra-line incompatibilities, but these will be immediately wiped out, as females bearing such *Wolbachia* will not be able to reproduce.

We established six isofemale lines for this study (two for each repertoire), in 2021. Repertoires have been stable since (last check in September 2023), i.e. for over two years or ~20 mosquito generations. Hatching rates for intra-crosses of all of these lines are now presented in Table S3.

2. I found the paper to be hard to follow and difficult to read.

We tried to simplify the text and significantly shortened the discussion, hoping it would make the paper clearer. We also improved the figures, which should make the reading easier.

A. The nomenclature/description of CI types/genes is unnecessarily complicated and inaccessible to someone outside of the Wolbachia-Culex field. I fear that these descriptions will scare readers away and I challenge the authors to make this more accessible.

Indeed, our nomenclature is complex reflecting the complexity of duplication and recombination that shapes *cidA* and *cidB* diversity! We added a part explaining the nomenclature in the Fig1, which should greatly improve the understanding (and we will keep in mind that this is necessary for future works, thanks!). We also wrote all the Greek letters in Greek rather than in Latin letters.

B. Figures 2 and 3 are not clear or visually pleasing. The nomenclature/description is difficult to follow. Also, I find that in these figures, the new lab mutant is lost among all of the other strains that are being considered.

We changed the figures and improved them visually. However, we were probably unclear in the previous version, and tried to improve the clarity of the key message, but yes, this variant is lost, not gained, explaining the figures.

C. The paper (especially the discussion) is too long and hard to follow. Many points of discussion can be shortened. Many of the sections do not need their own sub-headings. The hatching rate variation (and discussion) is confusing. This might be clearer if I had a better sense of what variation looks like when crossing females with males from the same line.

Intra-line hatching rates are now presented in Table S3.

The discussion of qPCR variation is confusing. As the authors recognize, it is difficult to disentangle technical artefacts from true variation, but their treatment and discussion of this is not so clear. The discussion of ruling out the possibility of multiple strains of Wolbachia is too long and not so clear. These long and sometimes confusing sections take away from key parts, such as the discussion of how this work connects to theory of CI evolution (i.e. Turelli 1994).

We shortened and clarified the “No sign of stable coinfection” section.

We also ran additional qPCR, and measured *cidA* copy numbers twice independently in the same (β+,16+) individuals. While we find that *cidA* copy numbers are always around five copies, we find no significant correlation between the first and the second measurements (Fig S7), demonstrating that variations around 5 copies are measurement errors rather than biological variation.

D. The writing can be strengthened for clarity. I found the use of the past tense to be confusing in places, because it is used to describe what was found both in the present study and in previous studies. Below are a few examples/suggestions where the wording is unclear and/or can be tightened up:

-Line 109. Change 'The cidA variant whose presence/absence best matches the CI variations has original aminoacid combinations at its interaction interface with CidB, probably resulting from a recombination.' to 'The cidA variant whose presence best explains the change in compatibility has a novel amino acid sequence at its interaction interface with CidB, probably resulting from a recombination event.'

Thanks, we used this sentence, except for it is not the presence but absence that explains the change in compatibility.

-Line 114. Change 'In 2021, crosses between Slab females and Ist males highlighted that some of the Slab females were fully incompatible with Ist males for the first time.' to 'In 2021, we found that some Slab females were fully incompatible with Ist males for the first time.'

Done.

-Line 146. I don't understand what 'we metonymically qualified mosquitoes' means.

It means we named the mosquitoes by the *Wolbachia* name, saying for instance (β+,16+) instead of “mosquitoes infected by a (β+,16+) *Wolbachia*”.

Reviewer #3:

This is a nice study from Namais and colleagues who found naturally occurring evolution of their lab colonies and used that to understand the mechanism by which Wolbachia-mediated CI functions and can evolve. This draft appears to be a revision, but I am seeing this for the first time, so I hope my comments are helpful, given this context. My major comments include some suggestions to simply and clarify the text given the density of information, and some requests for more details. Please see below for specific comments.

The draft appears as a revision because it went from a “regular submission” to a “short report” submission. However, it has never been reviewed before. Thanks for your comments, we tried to simplify and clarify the text.

Line 91 - presumably, the authors mean "in Wolbachia" here?

Yes, this was added for clarity, thanks!

Line 102-111: The authors mention in the introduction that they ruled out the contamination hypothesis, but I do not see the data directly used to infer this in the results (though I do see mention of this in the discussion).

The main argument is the fact that repertoires are highly variable among distinct strains (Bonneau et al, Nat Coms 2018, Mol Ecol 2019, Sicard et al, 2021), while sub-lines here only differ by the presence/absence of one or two variants, and share and identical architecture. For more clarity, the results section now presents first the similar organization, and the “lack of coinfection” part comes at the end. A sentence was added at the beginning of that section (starting line 280) to make that clearer.

Line 114-115: Is there data for this? What proportion of the crosses were compatible? What was the compatibility like prior to this? Not full I presume? Were there intermediate hatch rates here too? It would be great to see the pre- and post- 2017 crosses next to each other in one figure, if possible.

See the answers to reviewer #1: we made that part clearer (lines 111-117), by adding the references and explaining clearly which crosses were made for the current study, and which are previous results. We also gave more details on previous hatching rates.

There are quite a lot of abbreviations in the manuscript, and I found I regularly needed to refer back to earlier portions to keep track of which mosquito line was which, the cid variants, crossing directions, etc. For example, the key information that separates categories in figure 1 is in supplemental figure S1, which really interrupts reading/interpretation. Would it be possible to combine some of the figures to better link the genotypes/phenotypes? Or include a schematic that summarizes information from the introduction to show the cid operon with annotations for the important regions (upstream, downstream, cidA-beta,etc), which lines are compatible in which directions, etc?

Thanks, we changed the figures to make them clearer: (i) Figure 1 now combines the phenotypic and genotypic information, and (ii) Figure 2 & Figure 2 legend now contain a clear summary on overview of the information for *cidA* and *cidB*, along with a better explanation of the nomenclature.

We added “females” before the lines’ names in Figure 1, to clear the fact that females from these lines are crossed with Ist males. (In)compatibility of the crosses is now clearly stated in that figure.

Are there statistics for figure 1? (or for any of the other hatching rate comparisons?)

Statistics were added to the legend of the Figure 1.

Figure S2: quantification of Wolbachia titers: Is this adult female Wolbachia titer? After they laid eggs? I wonder if this is the most useful sample for determining the relationship between Wolbachia titer and hatch rate. For example, is Wolbachia titer in each embryo more important for whether or not that given embryo will hatch? Obviously some technical challenges when wanting to also determine hatch rate, but perhaps a note that whole-body titers of the mother might not be informative.

Yes, *Wolbachia* titers used are quantified in whole-body adult females, after they laid eggs. This is now mentioned in line 484. Indeed, *Wolbachia* titers in the embryos may have been more informative in terms of variability of HR in compatible crosses, we keep that in mind for future work!

Line 221: To clarify: were all 30 of these mosquitoes from 2005, or was 2005 just the earliest date?

All of them were from 2005 at the time the isofemale line was established, we tried to make that sentence clearer.

While the molecular methods are relatively standard, it would be best to include the reagents used for PCR/qPCR (e.g., enzyme/mastermix).

Reagents are now mentioned lines 477 and 482-483.

I would like to see more data reported on the nanopore sequencing results, especially because the authors are using coverage information to infer copy number. How many reads were produced for each PCR product? For statements such as line 274 "As these relative coverages were similar", please report the coverage data (total, and by cid). It looks like the method was previously published, but the authors might consider briefly discussing how sequences were compiled and how sequencing errors versus variants were called.

We made that part clearer in the section “Multiple copies of the same variants” in the Results, by adding that the average coverage for a given gene was between 30,000 and 50,000 reads. We also added a reference to our previous study, in which we showed that Nanopore sequencing coverage infers relative variant copy number (Namias et al, Computational and Structural Biology, 2023). Data showing that relative coverage are similar in both cases are now shown in lines 268-270.

Regarding copy number: are any of the cid operons in putatively functional prophages? Is there a possibility that some phage particles are being made and cid copy number measurements represent both Wolbachia and phage genomes? Or, perhaps a similar processes could be in play if replicative IS elements are copying and moving the regions around.

In the reference genome wPip-Pel, *cid* genes are located in prophage WO WO*w*Pip1, which has no tail modules suggesting no ability to get lytic (Klasson et al, 2008 and Bordenstein & Bordenstein, Plos Genetics 2022).

All *cid* copy number measurements are normalized on a single copy *Wolbachia* gene (*wsp*). If *cid* genes from free phage particles could be amplified, we would likely get huge number of *cid* copies, which is not what we observe, with copy numbers centered on 3 and 5.

My final comment is regarding Lines 54-62: While I agree with the authors' assessment that the TA model is a better fit, I do think the discussion of the models (of which there are now arguably three, because lines 40-42 discuss "mod-resc") detracts from the main points. This causes the manuscript to read as fixation on supporting a model, rather than trying to understand the root cause of the very interesting complexity in the wPip system. In general, the introduction is incredibly information dense (alas, by nature of the system), and it just isn't clear what the HM/TA model choice contributes to understanding the data/biology (and indeed, this is not revisited in the discussion). All this being said, I will leave it up to the editor/authors, but my suggestion would simplify, which will make the manuscript appeal to a much broader audience.

We changed that part and only explained the toxin-antidote model which fits better to *Culex* data. We kept the part explaining how the TA works, because it is important to understand what follows, which is: that losing a specific antidote results in a loss of compatibility. Following comments by the editor and other reviewers, we also made clear in the abstract and the text that this study was a clear argument for the TA model, and the first time that this model was demonstrated *in natura*, without using transgenes.

__________________________________________________

In compliance with data protection regulations, you may request that we remove your personal registration details at any time. (Use the following URL: <https://www.editorialmanager.com/pbiology/login.asp?a=r>). Please contact the publication office if you have any questions.
