# Supplementary material for: Intra-lineage microevolution of Wolbachia leads to the emergence of new cytoplasmic incompatibility patterns
Source: PLoS Biol. 2024 Feb 5;22(2):e3002493. doi: 10.1371/journal.pbio.3002493 (PMC10868858; doi:10.1371/journal.pbio.3002493)
Supplement: Attachment — Submitted filename: Answer_Reviewers_Namias_round2.docx [file pbio.3002493.s021.docx]

Dear Editor and Reviewers,

Thanks once more for your helpful insights. We followed your comments, added the required figures, and made the required text or figure changes.

Details about the changes made can be found below.

REVIEWERS' COMMENTS:

Reviewer #1:

The authors have addressed all my previous suggestions which has led to a substantially improved manuscript. I have no other major comments to make.

Thank you.

Reviewer #3:

Thanks to the authors for a careful and thorough revision of their manuscript. I maintain that this is a really interesting finding and the authors have done a lot of nice work to characterize the mechanisms responsible for these recently evolved CI patterns.

Thanks a lot.

Please see below for some minor comments to help with clarity. 

Line 23: "In wPip, CI is thought to function as a toxin-antidote (TA) system, with cidA
the antidotes, cidB the toxins and compatibility relying on having the right antidotes in the female to bind and neutralize the male's toxins"  -- this grammar reads oddly to me. Suggest:
"In wPip, CI is thought to function as a toxin-antidote (TA) system where compatibility relies on having the right antidotes (CidA) in the female to bind and neutralize the male's toxins (CidB)".

Thanks, we replaced that sentence by what was suggested.

Line 29 "original sequence at its binding interface, matching the original sequence at the toxin's binding" - is it the sequence that truly matches? Or just the shape of the protein?

It has been shown that the nature of the amino acids exposed at this interface influences the binding (Wang et al, Nat Com 2022). Their influence on the shape of the protein is not known. We replaced the word “matching” by the word “corresponding”.

I think there is still an opportunity to clarify the report of the initial key finding of incompatibility (lines 110-116). The sentences indicate 2021 data showing newly formed incompatibility (my assumption is that these have not yet been published, and the crosses were performed in 2021 - but I think the confusion is because the key Sicard et al paper was published in 2021). There are no data presented to accompany these details, and the reader has to infer the differences since 2017. I think it would be so much more impactful to re-plot the 2017 crosses next to the 2021 crosses. Finally, Line 116 "The hatching rates of the 69 compatible clutches varied between from 3.2% to 96.4%" - which crosses here are "compatible"? Or, these are the clutches from the newly incompatible cross in which at least some larvae hatched? I think it is confusing to call them compatible when the argument is that this cross is no longer compatible. If I am correct in my interpretation here, I
suggest:

In the present study (crosses performed in 2021), we witnessed for the first time the emergence of full incompatibly between some Slab females and Ist males. Twenty five of 94 rafts had a 0% hatch rate, indicating strong CI, and the remaining 69 clutches had hatch rates between 3.2% to 96.4%.

Thanks for pointing out the potential confusion with the Sicard et al 2021 paper. We used the sentence suggested above to describe our data, and also tried to clarify the explanations for the Sicard et al paper (line 112).

As suggested, we plotted the hatching rates distribution in 2017 and in 2021 in a new supplementary Figure (Figure S1) to make the differences clearer.

Line 142: "specifically screened the 89 females" - the previous section discussing the females 102 females, 94 of which were mated. It is unclear what the 89 is referring to.

Thanks, that was not clear indeed! We screened the 94 mated females, but out of those only 89 were successfully screened by qPCR. We made that clear.

Line 147: I agree that with the other reviewer that the phrase "metonymically qualified mosquitoes" is jargon - I also agree with the authors response that it makes sense to do this, but perhaps consider editing the manuscript to instead state "hereinafter we refer to a mosquitos by its Wolbachia infections' cidA genotype repertoire (e.g., (β+,16+), (β+,16-), or (β-,16-)"

We used the sentence suggested, thanks!

Line 310 "Our microevolution approach is another functional strategy, additional to transgene expression…" - I agree with the conclusion that the data support T/A but, it does seem unusual to call it "your approach", as the authors were more very lucky and very observant to catch this phenomenon… rather than intentionally evolving lines experimentally.

That is true! We were lucky to observe this phenomenon. However, we had shown that CI patterns could evolve for a long time, but did not have the tools to identify the genomic basis of these changes and rule out contamination. This observation of long-term changes in CI patterns would not have been possible without the long-term rearing which we established, associated with regular crosses between the lines maintained at the lab. We changed the sentence in that sense.

Some of the figures use a combination of red and green, which is likely not colorblind friendly.

That is true, thanks for noticing, we used colorblind-friendly palettes for the new figures created, and changed colors of the other figures to ensure that red and green did not coexist in a given figure.


COMMENTS FROM THE ACADEMIC EDITOR:

I would add that in line 123 the reference should be to another figure or table, not S1 Fig.

Thanks a lot, we meant to refer to Table S1 indeed!

Also, I think that figure S2 should be included in figure 2. This is the clearest visualization of the relationship between recombination, sequence, and compatibility phenotypes.

We integrated Figure S2 in Figure 2.

Finally, the data on hatching rates present in many of the tables in supplementary information should be represented in supplementary figures too.

Data in Supplementary Table S2 (now renamed Data S1) is already represented in Figures S1, S3, and S6. Data S2, S3 and S4 (previously Supplementary Tables) are now represented in new Supplementary Figures (Figs S3-5).
